# What Contributes to Student Language Learning Satisfaction and Achievement with Learning Management Systems?

**DOI:** 10.3390/bs14040271

**Published:** 2024-03-24

**Authors:** Hanxue Li, Aohua Ni

**Affiliations:** 1 College of Education, Hunan First Normal University, Changsha 410205, China; 2010201160008@whu.edu.cn; 2 Graduate School of Education, Peking University, Beijing 100871, China

**Keywords:** e-learning, learning management system, learning satisfaction, achievement, English language learning, secondary education

## Abstract

Learning management systems (LMSs) have received substantial global attention and have undergone extensive research, with most discussions focusing on users’ acceptance and continuation of LMS use in the higher education sector. However, research is scarce in terms of identifying the factors that are advantageous to K-12 students’ learning and satisfaction when using LMSs for language learning. This study aims to examine the impacts of internal and contextual factors on secondary students’ learning satisfaction and English achievement when using LMSs. Data were collected from 289 students through an online survey. The results of the structural equation modeling showed that satisfaction had the most significant impact on English achievement. Furthermore, both internal and contextual factors, including technology self-efficacy, interest, task value, teacher support, and technology facilitation, positively impacted learning satisfaction with LMSs. In addition, teacher support exerted the strongest impact on satisfaction, followed by interest and technology self-efficacy. However, only internal factors, such as interest and task value, were positively associated with English achievement. Neither teacher support nor technology facilitation significantly impacted English performance. Given the increasing availability of LMS usage, the findings of this study can facilitate the more effective implementation of LMSs in China and globally. The study contributes to the theory and practice of LMSs use in K-12 English education. The limitations and implications of the study were discussed as well.

## 1. Introduction

Educators have increasingly advocated e-learning as a promising alternative to the traditional face-to-face learning approach, primarily due to its advantages of enhanced learning efficiency and effectiveness and its ability to overcome limitations of time and space [1,2]. The COVID-19 pandemic has further solidified e-learning as an unavoidable and irreversible trend in the global evolution of education [3]. One important e-learning tool is a learning management system (LMS), which functions as a web-based learning environment that offers a virtual platform for both asynchronous and synchronous learning. This platform allows teachers and students to access learning materials, engage in collaborative discussions, receive feedback, and facilitate personalized learning [4]. There are many LMSs used in the education sector, for example, Moodle, which is currently utilized in 246 countries, boasting an impressive user base of 270 million and 36 million courses [5]. Through Moodle, students can access course materials and complete the modules and assignments at their own pace, while also using features such as instant messaging and discussion forums. Additionally, education institutions and instructors can efficiently manage and deliver online courses while tracking students’ progress. The use of LMSs has made significant contributions to language education and has gained even more attention in the post-pandemic era [6]. LMSs provide a wide range of adaptable resources, including reading materials, interactive tasks, and multimedia content for language learners of various learning styles and linguistic backgrounds [7]. Moreover, empirical studies have demonstrated the widespread agreement among researchers and educators regarding the positive effects of LMSs on cognitive development, academic achievement, engagement, motivation, as well as the provision of productive learning experiences for language learners [2,8,9,10,11]. Therefore, to optimize the use of LMSs in language education and improve learning outcomes, it is crucial to understand the factors that influence students’ learning experiences when using LMSs.

Along with their fast growth, LMSs have gained substantial global attention and have undergone extensive research. However, the discussions have mainly focused on users’ acceptance and continuation of LMS use [1,4,5,12,13]. In recent years, e-learning tools, including LMSs, have become increasingly common in blended or hybrid learning contexts, owing to the penetration of information and communication technology in pedagogical practices [4]. Notably, in China, the e-learning industry has demonstrated steady growth, with the industry size reaching CNY 485.8 billion (nearly USD 71 billion) in 2020, representing a year-over-year rise of 20.2% (Forward-The-Economist. (17 March 2022). *Panorama of China’s online education industry in 2022*. Retrieved 8 May 2022, from https://www.qianzhan.com/analyst/detail/220/220317-72b3ce09.html). In China, LMSs have witnessed a high rate of usage in elementary and secondary education [4]. Thus, given the wide availability of LMS usage in Chinese education systems, the question now pertains to improving students’ learning experiences and outcomes when using LMSs, rather than their acceptance of LMSs. Addressing this question can facilitate the more effective implementation of LMSs not only in China, but also globally. 

Recent research trends have shifted towards exploring the factors that contribute to the success of e-learning [14,15]. However, a majority of these studies have focused on higher education, with few exploring the K-12 education setting [15,16]. Specifically, there is a scarcity of research on identifying the factors that are advantageous to K-12 students’ learning and satisfaction when using LMSs for language learning. Previous research has demonstrated the significance of contextual and environmental factors, such as technical assistance, Internet access, and instructional quality, in students’ e-learning adaptation and readiness [17,18]. Moreover, individual or internal factors, such as interest, enjoyment, and self-efficacy, have been identified as important motivational and psychological predictors of e-learning experiences and achievement [19,20]. However, while contextual factors have been more widely studied in relation to students’ e-learning satisfaction, the impact of intra-individual factors, such as motivational beliefs, has not been thoroughly explored [21]. Consequently, it remains unclear how internal factors influence K-12 language learners’ satisfaction and learning outcomes with LMSs, and this demands further investigation.

The present study addresses the research gaps by incorporating internal and contextual factors and examining their impact on secondary students’ learning satisfaction and English achievement when using LMSs. By integrating multiple factors into a single study, this research offers new insights into the existing literature on LMSs, thereby expanding the current understanding of technology acceptance to encompass learning experiences. Given the current global trend of e-learning, this study has significant implications for implementing LMSs in language education, providing valuable guidance for researchers and practitioners seeking to integrate LMSs into secondary language instruction successfully.

## 2. Literature Review

### 2.1. Learning Management System (LMS)

LMSs, as an e-learning tool, encompass a type of management software and integrated system that aims to facilitate teaching and learning in a structured manner by managing resources, customizing the learning process, monitoring interactions, and evaluating performance [22]. Prior research has identified four fundamental components of LMSs: “content management”, which involves uploading and downloading resources; “user account management”, which involves building a database containing detailed information about the users; “communication”, which uses chat rooms or forums for interaction; and “evaluation”, which includes tasks such as homework and quizzes for assessment purposes [7]. These four components reveal several distinct features of LMSs. First, LMSs serve a “pedagogical” purpose by transferring learning content to the system and tracking learning processes and achievement levels [6]. Second, LMSs create a highly “interactive” environment by employing synchronous and asynchronous communication tools for discussion and feedback [23]. Third, LMSs are used “systematically” to make announcements, manage course arrangements, and propose and collect assignments within the given timeframe [22]. Such characteristics render LMSs advantageous in terms of convenience, flexibility, and interactivity. Based on their features, LMSs have been defined from multiple perspectives, including the instructor’s viewpoint and the learner-centered perspective, as a means to cater to the growing trend of active learning [24]. In the context of language learning, the role of LMSs has become increasingly prominent, particularly in the last decade and even more so after the pandemic period due to increased access to the Internet and advancements in language teaching and learning technologies [25]. Currently, there are 561 LMSs available worldwide for academic purposes, with popular ones such as Moodle, MOOC, Edmodo, and Google Classroom dominating the market [11]. The widespread adoption and exponential growth of LMSs globally have solidified their importance in academia.

### 2.2. The Framework of e-Learning Success

**Figure 1:**
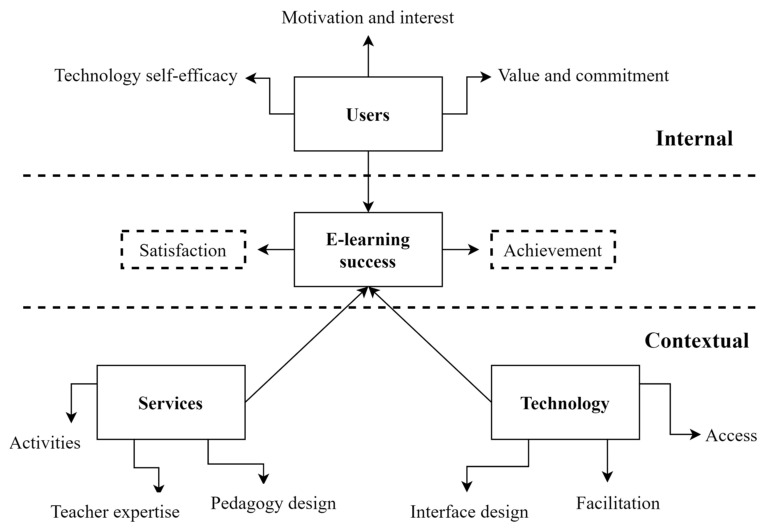
An analytical framework for e-learning integration in LMSs.

In response to the growing importance of e-learning, educational institutions worldwide increasingly emphasize the need for sustainable e-learning initiatives to improve learning readiness and outcomes. Previous research has identified a range of factors contributing to e-learning success. Aparicio et al. [26] synthesized various scopes of e-learning studies and proposed a theoretical e-learning framework including three principal aspects, “users”, “technology”, and “services”, to build an e-learning ecosystem. The paradigm acknowledges that individuals use e-learning systems, that technology facilitates varied user engagement (directly and indirectly), and that services incorporate all pedagogical models and instructional activities. This study applies Aparicio et al.’s holistic framework of e-learning to analyze the factors influencing the use of LMSs. We extended the framework to include two aspects, “internal” and “contextual”, of holistic e-learning systems to examine the factors relevant to LMS use. Internal factors relate to the learner-related aspects that reflect individual characteristics and inner motivational states of e-learners, which impact their perceived values and experiences in e-learning, as well as their satisfaction and learning outcomes [3]. Contextual factors in e-learning refer to situational or environmental characteristics that may impact students’ technology adoption behaviors, learning experiences, and outcomes [27]. For the first perspective, “internal”, the effectiveness evaluation was conducted regarding students from the “users” dimension, which focused on students’ interest and confidence in using LMSs for English learning. The second perspective was the “contextual” dimension, which examined how teachers supported students in LMSs learning and the technological facilitation available to students for LMS use. Figure 1 depicts the theoretical model applied in this study, which builds on Aparicio et al.’s framework and extends it to analyze the factors influencing LMS use in English learning.

### 2.3. Satisfaction and Achievement in e-Learning

Satisfaction has been widely recognized as the ultimate objective of any product or service and as one of the most widely accepted metrics used to assess the quality and effectiveness of teaching and learning methods [27]. It is a subjective psychological state that encompasses positive emotions and behavioral outcomes stemming from the successful completion of desired learning activities or processes [3]. In the present study, satisfaction was defined as secondary students’ evaluation of their learning experiences when using LMSs to learn English, as reflected in their perceptions of the benefits gained from this technology [15]. In the context of e-learning, satisfaction is influenced by the cognitive and emotional gap between learners’ actual perceived benefits and their expectations [3]. 

Satisfaction plays a critical role in determining the effectiveness of e-learning in terms of student learning behaviors and outcomes. Previous studies have indicated that learning satisfaction significantly predicts students’ continued use of e-learning tools, including LMSs [18,28]. In addition, as a psychological response, satisfaction with e-learning is positively associated with motivational levels, engagement, self-efficacy, persistence, and perceived learning outcomes [20,29,30]. As a result of its demonstrated positive correlation with the quality of learning outcomes, learning satisfaction has come to be recognized as a crucial indication of e-learning success [21,23]. For example, Dinh and Nguyen [31] found that Viennese students who reported greater satisfaction with e-learning achieved higher academic performance. Therefore, we propose the following hypothesis.

**H1.**  *Learning satisfaction positively influences secondary students’ English achievement when using LMSs.*

### 2.4. Internal Factors

Satisfaction has emerged as a critical variable in e-learning research, given its impact on educational outcomes [14]. To better understand the drivers of satisfaction and learning outcomes in e-learning, past research has examined various factors, including learners’ perceived value or the usefulness of e-learning [23], self-efficacy, self-motivation, interest [32], perceptions of the teachers [29], the design of course content and structure [3], and the platform quality and technical support services [27] These factors can be classified as either internal (e.g., learner characteristics) or contextual (e.g., course design, platform quality) to exert influences on learning satisfaction and outcomes. 

Internal factors refer to the learner-related aspects that represent the individual characteristics and inner motivational states of e-learners, which influence their perceived values and experiences in e-learning, as well as their satisfaction and learning outcomes [3]. Motivation has been established as a key factor in the success of e-learning, which underscores the importance of examining how motivational beliefs impact student satisfaction and achievement in K-12 English education when using LMSs [6]. This study focused on self-efficacy, interest, and perceived task value as internal factors to investigate their relationships with students’ satisfaction and English performance. 

#### 2.4.1. Technology Self-Efficacy

Self-efficacy is a key component of social cognitive theory, which influences human agency by affecting activity selection, effort, and accomplishment [33]. The present study defines self-efficacy as the confidence of secondary students in their capacity to complete English learning activities effectively when using LMSs [34]. A number of existing studies have indicated a significant positive relationship between self-efficacy and learning satisfaction in e-learning contexts [20,35]. For example, Alqurashi [36] examined 167 e-learners and found that self-efficacy was the strongest predictor of perceived learning outcomes and a significant antecedent of satisfaction. In English language learning, empirical research has also revealed that self-efficacy is a crucial predictor of academic achievement [37]. Based on these findings, it is reasonable to assume that self-efficacy will continue to positively impact English learning when using LMSs. Therefore, we propose the following hypotheses.

**H2a.**  *Technology self-efficacy positively and directly influences students’ learning satisfaction toward LMSs.*

**H2b.**  *Technology self-efficacy positively and directly influences students’ English achievement.*

#### 2.4.2. Interest

Intrinsic motivation, such as interest, is a significant factor influencing e-learning behaviors, as it reflects the desire to engage in online learning for the inherent pleasure and enjoyment of the activity rather than for external rewards or academic performance consequences [5]. Prior studies have demonstrated that interest is the most important educational input and intrinsic motivator for e-learning behavior [38]. According to the expectancy–value theory, students are intrinsically driven to learn when they experience happiness and enjoyment during the learning process [39]. Interest and enjoyment have been found to significantly impact both learning satisfaction and academic performance in e-learning contexts [17,29,40]. Thus, we propose the following hypotheses.

**H3a.**  *Interest positively and directly influences students’ learning satisfaction toward LMSs.*

**H3b.**  *Interest positively and directly influences students’ English achievement.*

#### 2.4.3. Task Value

Perceived task value refers to students’ internal evaluation of the usefulness and value of e-learning, reflecting the extent to which secondary students believe that e-learning activities can help improve their English performance [27]. Previous research has highlighted the importance of perceived task value in academic achievement by examining its positive association with learning satisfaction [13,27]. According to the expectancy–value theory, learners’ satisfaction greatly depends on the value they place on a given task [40]. This is crucial, since a well-established connection exists between satisfaction and achievement outcomes [23,29]. When students realize the benefits of using LMSs for English learning, they are more likely to be satisfied and invest more efforts into learning activities, resulting in improved learning outcomes. Based on this, we developed the following hypotheses.

**H4a.**  *Task value positively and directly influences students’ learning satisfaction toward LMSs.*

**H4b.**  *Task value positively and directly influences students’ English achievement.*

### 2.5. Contextual Factors

Contextual factors in e-learning refer to situational or environmental characteristics that may impact students’ technology adoption behavior, learning experiences, and outcomes. A rising corpus of research has emphasized the significance of environment and context in students’ e-learning experiences and outcomes [27,30]. To examine the usability of LMSs and the extent to which students receive assistance from their teachers, this study used technology facilitation and teacher support as contextual factors.

#### 2.5.1. Technology Facilitation

Technology facilitation, which refers to the stability of LMSs and the technical support students receive, plays a crucial role in the system’s usability and the quality of LMSs [27]. The technology acceptance model (TAM) suggests that students’ perceptions of the accessibility and quality of technical services they receive significantly influence their continuous intention to use LMSs [22]. Based on TAM, numerous studies have provided convincing evidence for the importance of facilitating conditions, such as stable Internet access and good service quality, in making LMSs easy to use, which, consequently, are a significant predictor of student satisfaction and learning outcomes [17,20,23]. Therefore, we propose the following hypotheses.

**H5a.**  *Technology facilitation positively and directly influences students’ learning satisfaction toward LMSs.*

**H5b.**  *Technology facilitation positively and directly influences students’ English achievement.*

#### 2.5.2. Teacher Support

Teacher support in this study refers to the assistance and instructional guidance provided by teachers via LMSs, including the provision of high-quality course materials, timely feedback, and active interaction with students. Prior research has established that teachers’ instrumental, appraisal, and emotional assistance significantly impact students’ satisfaction and learning achievement, regardless of the learning setting, whether it is traditional or online [3,28,29]. Teachers’ knowledge and expertise, attitudes toward e-learning, teaching abilities, levels of interaction, and social presence all play critical roles in determining learners’ satisfaction with e-learning [21]. For example, a study of British students found that teacher-related factors—for example, providing e-learning resources and direct contact time with students—were the most influential factors affecting student satisfaction [41]. Therefore, based on the existing literature, we propose the following hypotheses and present the conceptual framework depicted in Figure 1.

**H6a.**  *Teacher support positively and directly influences students’ learning satisfaction toward LMSs.*

**H6b.**  *Teacher support positively and directly influences students’ English achievement.*

## 3. Method

### 3.1. Participants and Data Collection

The study used a convenient sampling method to recruit participants from a specific population. Grade 11 students from two public senior secondary schools in Central China were invited to take part in the study. The two schools are located within the same district and have comparable socioeconomic statuses. Both schools are top schools in the district and have a similar admission score lines. Students in the district come from similar family backgrounds, with an average household income of around CNY 130,000. The district implemented a free LMS called “Class Manager (CM)” in the schools in 2020 to better facilitate teaching and learning. At the time of the study, the participants had one year of experience using the LMS for English learning. 

CM is a typical LMS accessible to students through electronic devices like pads, tablets, or smartphones. CM has been designed to facilitate English language learning by dividing the process into three stages: before, in, and after. The before-class stage involves teachers making announcements and uploading course materials onto the system. Students need to view the materials and complete a quiz to ensure they are adequately prepared for the upcoming class. Teachers can track students’ progress and quiz completion in the system backstage. In the in-class stage, the roll call and group formation system is utilized to foster cooperative learning. In the after-class stage, teachers use the system to assign homework and evaluate student assignments. Additionally, students are expected to upload their speaking and writing tasks to the forum, where members of their learning groups are responsible for reviewing and proposing suggestions for improvement.

In November 2021, data were collected via a web-based questionnaire survey distributed to participants through school administrators. Prior to the pandemic, LMSs were not widely used in the district. However, with the emergence of the COVID-19 pandemic, the two schools in our study began to incorporate CM for teaching and learning. Schools had to adopt virtual platforms to align with the current trend of e-learning. Despite the challenges posed by the pandemic, the use of CM has yielded numerous benefits for students, instructors, and schools. Accordingly, they continued to implement CM even after the pandemic. Our study, which was conducted in 2021, immediately following the pandemic, provides valuable insights into how to effectively facilitate students’ learning with LMSs in the post-pandemic period. We chose grade 11 students from the two public schools as our participants for several reasons. Firstly, these schools share comparable socioeconomic statuses. Secondly, grade 11 students had had one year of experience with CM, enabling them to better comprehend the survey’s purpose and provide their own insights on their experiences with the CM system. Lastly, grade 11 students were not under the pressure of China’s College Entrance Examination as they were not in their final year of high school, allowing them to dedicate time to participate in the research. The survey was conducted after the mid-term examination, which was a district-level exam. Official English test scores were obtained from the two schools to evaluate English achievement. A total of 326 grade 11 students voluntarily participated in the survey, of which 289 submissions were deemed effective after data screening. Respondents who completed the survey in less than 180 s or provided meaningless information were excluded from the analysis. Thus, 37 unengaged respondents were identified and removed. There were no missing data since participants had to complete all questions before submission. Of the 289 participants, 48.4% (*n* = 140) were male students, and 51.6% (*n* = 149) were female. The average age of participants was 16.6 years old. Before conducting the survey, the study was ethically approved by the corresponding author’s institution.

### 3.2. Instrument

The study used a self-report questionnaire. Based on previous literature, the questionnaire was developed to fit the research hypotheses in the study. This 26-item scale was constructed with a 6-point Likert-type response format consisting of values ranging from “1” (strongly disagree) to “6” (strongly agree). The authors translated the scales’ items into Chinese and then back into English. Three secondary school students were asked to pre-fill the questionnaire for content validity.

For individual factors, technology self-efficacy (e.g., I am skilled at using LMS even if there is no one around to show me how to do it), interest (e.g., LMS makes learning English more enjoyable), and task value (e.g., I think LMS is useful for my English learning) were used with reference to the measurement items of previous studies that are applicable to the present study in China [6,13,38,42]. The Cronbach’s α values of technology self-efficacy, interest, and task value were 0.941, 0.958, and 0.946, respectively, showing good internal consistency. For contextual factors, three items used in Qin [43] (e.g., technology support is always available when using LMS) were revised to reflect technology facilitation. Five items selected and adapted from Cheng [44] and Diep [21] (e.g., my English teachers have high levels of expertise in teaching e-courses) were used to measure the level of students’ perceived teacher support in English e-classes. Additionally, the authors used four items to measure students’ satisfaction levels (e.g., I am satisfied with the effectiveness of LMS in assisting my English learning) by considering and incorporating previous models [12]. The Cronbach’s α values of technology facilitation, teacher support, and learning satisfaction were 0.932, 0.943, and 0.957, respectively, showing good internal consistency. In terms of achievement, English achievement was determined by the students’ mid-term scores, which were provided by the schools, with a maximum score of 150.

### 3.3. Data Analysis

All data analyses were performed in the SPSS and Mplus programs for analysis. We first checked the normality and correlation of the variables. Given the theoretical backing and extensive prior research on the scales used in this study, we conducted the confirmatory factor analysis (CFA) to evaluate the measurement model, including the construct reliability, validity, and measurement model fit, and checked the Cronbach’s α, factor loadings, composite reliability (CR), and average variance extracted (AVE). We then performed a structural equation model (SEM) to test the hypotheses and used multiple indices for the model fitness.

## 4. Results

Table 1 presents the descriptive statistics for the variable. All skewness and kurtosis values were less than one, which suggested that the data were normally distributed (Kline, 2015). Table 2 shows the correlation analysis and the discriminant validity assessment. All the factors were significantly associated with each other. The results also demonstrated good discriminant validity (values in bold).

### 4.1. The Measurement Model

CFA was conducted to examine the measurement model. The present study used the CFA fit criteria (χ^2^/df < 5, CFI > 0.90, TLI > 0.90, RMSEA < 0.08, SRMR < 0.08) based on previous recommendations of goodness-of-fit indices [45,46]. The path analysis was performed using a structural equation model (SEM), with bootstrapping performed on 500 subsamples after evaluating the reliability and validity. The measurement model fit was excellent: χ^2^/df = 1.323 (*p* < 0.001), CFI = 0.977, TLI = 0.974, RMSEA = 0.033, SRMR = 0.026. As presented in Table 3, all the values of CR exceeded the cut-off value of 0.70, and all the outcomes of AVE were above the cut-off value of 0.70 [47], showing good convergent validity.

### 4.2. Structural Model Analysis

**Figure 2:**
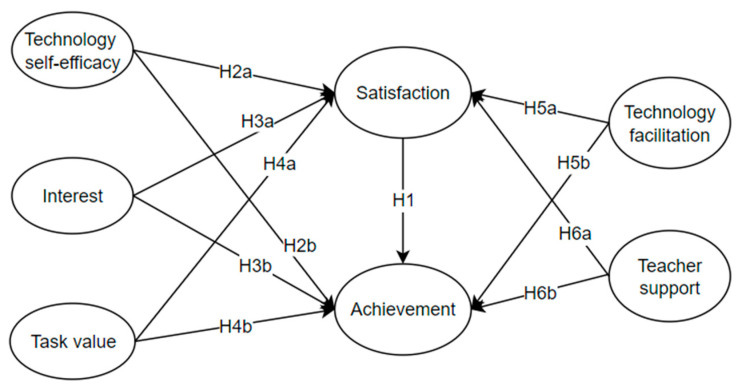
Proposed model.

In the present study, path analysis was conducted to examine the factors influencing secondary students’ learning satisfaction and English achievement when using LMSs. The fundamental statistics suggested a satisfactory structural model fit: χ^2^/df = 2.543 (*p* < 0.001), CFI = 0.949, TLI = 0.941, RMSEA = 0.073, SRMR = 0.025. Figure 2 shows the links among the variables, and the structural model results are presented in Table 4.

**Figure 3:**
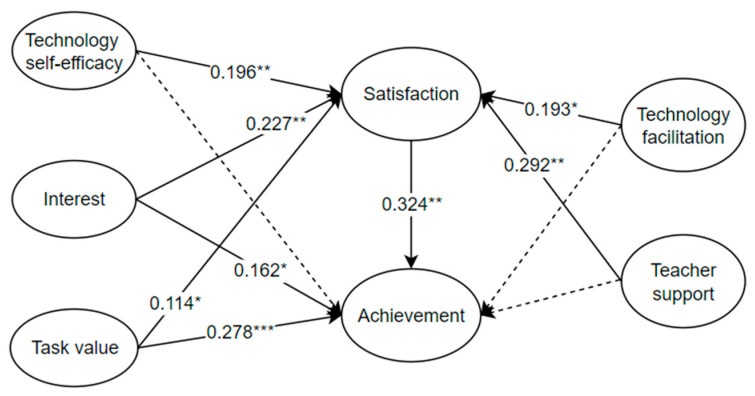
Path analysis for the model. * *p* < 0.05, ** *p* < 0.01, *** *p* < 0.001.

The analysis shows that the model explained 54.8% of the variance in secondary students’ English performance. Figure 3 shows the links among the variables in the structural model. It indicated that interest (*β* = 0.236, *p* < 0.01), task value (*β* = 0.315, *p* < 0.001), and learning satisfaction (*β* = 0.324, *p* < 0.01) were positively associated with English achievement, demonstrating that H1, H3b, and H4b were supported. The most influential factor contributing to English achievement was students’ learning satisfaction towards LMSs, followed by perceived task value and interest. However, among the individual factors, no association was found between students’ perceived technology self-efficacy and English achievement (*β* = 0.032, *p* > 0.05). Thus, H2b was rejected. As for the contextual aspect, technology facilitation had no impact on English achievement. Furthermore, teacher support only had an indirect effect on achievement. No direct positive association was found between teacher support and English achievement (*β* = 0.095, *p* < 0.05). Thus, H6a and H6b were not supported. 

Concerning the impact on learning satisfaction, teacher support demonstrated the strongest correlation with students’ learning satisfaction (*β* = 0.292, *p* < 0.01), followed by interest (*β* = 0.227, *p* < 0.05), supporting H6a and H3a. Technology self-efficacy was the third factor directly impacting students’ satisfaction (*β* = 0.196, *p* < 0.05), supporting H2a. In addition, technology facilitation (*β* = 0.193, *p* < 0.05) and perceived task value (*β* = 0.114, *p* < 0.05) were found to predict secondary students’ English achievement significantly positively when using LMSs, demonstrating that H5a and H4a were supported.

## 5. Discussion

Using a prominently proposed framework that combined learner-internal and contextual factors, the present study presents a model depicting the key guiding factors that influence secondary students’ learning satisfaction and English achievement when using LMSs. The majority of the hypotheses supported the general portrayal of the model. The confirmed aspects contributing to student learning satisfaction toward LMSs were technology self-efficacy, interest, perceived task value, teacher support, and technology facilitation. However, in terms of English learning outcomes, only satisfaction, interest, and task value significantly positively affected students’ academic performance.

Consistent with prior literature, the study confirmed that both learner-internal and contextual factors were significant determinants of the learning experiences and satisfaction with LMSs [3,27,29,41]. Regarding contextual factors, teacher support was found to have the highest direct effect on satisfaction (*β* = 0.292, *p* < 0.01). This result supported the findings of Langan and Harris [48], who analyzed 1.8 million student responses and reported that teacher- and teaching-related factors were more influential than learner factors in determining learning satisfaction. This is particularly important in the context of e-learning environments that lack face-to-face communications, where teachers’ responses and feedback are considerably more important to maintaining involvement [36]. Secondary students who have frequent contact with their English teachers and receive high-quality e-knowledge delivery are likelier to report high student satisfaction and learning gains. Therefore, it is essential for English teachers to adapt their classes to their students’ personalities and preferred learning styles, and to provide timely feedback and encouragement.

In addition, for contextual factors, it was found that there existed a direct positive relationship between technology facilitation and learning satisfaction. The finding was consistent with the results of numerous previous studies [27,29,49]. The finding emphasized the importance of technical support services and organizational assistance in enhancing students’ satisfaction with LMSs in English learning. Therefore, it is essential for schools to provide the necessary technological resources, staff support, and other forms of assistance to facilitate the implementation of LMSs and mitigate any potential obstacles that may arise.

As for learner-internal factors, the findings that technology self-efficacy, interest, and task value positively influenced students’ learning satisfaction echoed previous research [3,15,20,27]. The results suggest that students who used LMSs with a strong belief in their capacity to complete learning activities and fulfill course requirements successfully were more satisfied with their use of LMSs for English learning. The findings also underlined the importance of motivation in enhancing learning satisfaction [17]. When students have a greater interest in learning English using LMSs and a strong belief that LMSs will improve their English abilities, their learning satisfaction and effectiveness are likely to increase correspondingly. As a result, efforts should be invested to enhance students’ motivations and interest in using LMSs. English teachers need to create more appealing online learning tasks and provide more cooperative activities to increase student engagement. Additionally, to boost students’ self-efficacy, English teachers may consider providing more performance accomplishments and prompt, constructive feedback with encouragement.

In terms of English achievement, learning satisfaction toward LMSs was found to be the most critical predictor of student success. The finding was not surprising, given that numerous studies have demonstrated that student satisfaction has a direct bearing on their learning gains [21,23,31]. However, only learner-internal factors, such as students’ levels of interest and their perceptions of the value of LMSs, were found to be positively associated with secondary students’ English performance. Regarding contextual factors, the findings revealed that technology facilitation had no significant impact on English achievement. Moreover, teacher support only indirectly affected students’ English performance through the mediation of student satisfaction. The findings highlight the significance of motivation, which has consistently been shown to be crucial for e-learning success [38,50]. Students’ intention to learn is motivated by their inner expectations of achieving satisfactory outcomes and gaining improvement [5].

## 6. Conclusions and Limitations

The present study addressed LMS, a fast-growing e-learning tool, and attempted to understand how internal (technology self-efficacy, interest, and task value) and contextual (teacher support and technology facilitation) factors predict secondary students’ learning satisfaction and English achievement with the use of LMSs. The proposed model had good predictive power, explaining 54.8% of the total variance. 

The present study has theoretical and practical implications for using LMSs in language education. From a theoretical perspective, this study contributes to the existing body of knowledge by providing empirical evidence to support a suggested model for the effective LMS use of LMSs in language education. The model has demonstrated strong predictive power, suggesting that it may have broader theoretical implications for future research into the effectiveness of LMSs in language education. From a practical standpoint, this study confirms previous findings on the factors influencing e-learning satisfaction. It highlights the importance of contextual and internal factors for successfully implementing LMSs in language education. Specifically, the study identifies satisfaction as the factor that has the most significant impact on learning achievement. Therefore, improving LMS-facilitating conditions, such as creating a more user-friendly interface, providing timely user response service, and enhancing instructor expertise, including academic metrics and digital competence, can increase learning satisfaction. Specifically, to ensure proficiency in using LMS, schools and educators should provide comprehensive training and ongoing support to students and teachers, including workshops, tutorials, and resources to help users navigate the system effectively. LMS developers should focus on creating user-friendly interfaces that are intuitive and visually appealing. Clear instructions and well-organized course materials can improve the overall user experience, resulting in higher student satisfaction and engagement. Additionally, the study highlights the importance of motivational beliefs for successfully using LMSs in language education. To increase students’ interest in using LMSs, teachers should design engaging and interactive learning tasks within the LMS that promote collaboration among students, such as group projects, discussions, and multimedia-rich content. Continuous innovation by LMS developers is crucial to enhance the learning experience. Introducing new features, such as gamification elements, multimedia integration, and personalized learning paths, can increase student engagement and satisfaction with the LMS. Furthermore, it is crucial for students to recognize the value of LMSs in facilitating English language learning. Thus, schools, teachers, and system developers need to publicize the advantages of LMSs for English language learning, emphasizing the benefits of using LMSs in language education.

There are some limitations to be noted. First, the sample size was small, which may limit the generalizability of the results. A larger and more representative sample size may be considered in future studies. Second, the data collection relied on self-reported measures, which may have resulted in self-reported bias threatening the results’ reliability. Third, even though the study aimed to determine how various internal and contextual variables influenced students’ online learning satisfaction and English achievement, we must realize that other variables may have also played a role, but were not included in the analysis. For example, factors such as transferability, efficiency, effectiveness, and collaboration may also impact students’ satisfaction and achievements in online learning. Therefore, potential directions for future research could involve exploring these additional key elements and incorporating qualitative research to gain a deeper understanding of their relevance. Finally, the study employed cross-sectional data for analysis. Future research may consider using experimental designs to explain causality and incorporating qualitative approaches to better understand students’ learning experiences with LMSs.

## Figures and Tables

**Table 1:** Descriptive statistics.

Factors	Mean	SD	Skewness	Kurtosis
Technology self-efficacy	4.307	1.015	−0.592	0.293
Interest	4.230	1.178	−0.630	−0.094
Task value	3.748	1.624	−0.253	−0.383
Technology facilitation	4.246	1.201	−0.554	−0.270
Teacher support	4.429	1.091	−0.805	0.451
Satisfaction	4.263	1.123	−0.657	0.020
Achievement	89.746	19.276	−0.395	0.204

**Table 2:** Correlation analysis of variables.

Factors	TSE	INT	TSV	TF	TS	SA	ACH
Technology self-efficacy	**0.875**						
Interest	0.593 **	**0.907**					
Task value	0.373 **	0.624 **	**0.902**				
Technology facilitation	0.560 **	0.642 **	0.570 **	**0.907**			
Teacher support	0.655 **	0.685 **	0.573 **	0.771 **	**0.878**		
Satisfaction	0.663 **	0.735 **	0.608 **	0.732 **	0.773 **	**0.921**	
Achievement	0.451 **	0.628 **	0.614 **	0.590 **	0.594 **	0.672 **	-

** *p* < 0.01.

**Table 3:** Measurement model.

Measures	Items	Factor Loading	CR	AVE
Technology self-efficacy	5	0.854–0.915	0.942	0.765
Interest	5	0.873–0.941	0.959	0.824
Task value	4	0.879–0.920	0.946	0.814
Technology facilitation	3	0.872–0.941	0.933	0.824
Teacher support	5	0.772–0.911	0.943	0.772
Satisfaction	4	0.887–0.961	0.958	0.850

**Table 4:** Results of the structural model.

	Satisfaction	Achievement		
	Direct Effects	Direct Effects	Indirect Effects	Total Effects
Technology self-efficacy	0.196 **	−0.031	0.063	0.032
Interest	0.227 **	0.162 *	0.074 *	0.236 **
Task value	0.114 *	0.278 ***	0.037 *	0.315 ***
Technology facilitation	0.193 *	0.085	0.063	0.147
Teacher support	0.292 **	0.018	0.095 *	0.112
Satisfaction	-	0.324 **	-	0.324 **
R^2^	-	0.548 ***	-	-

* *p* < 0.05, ** *p* < 0.01, *** *p* < 0.001.

## Data Availability

Tables include show detailed information of the analyzed data.
